# Supplementary material for: Applying citizen science to engage families affected by ovarian cancer in developing genetic service outreach strategies
Source: PLoS One. 2022 Feb 14;17(2):e0262575. doi: 10.1371/journal.pone.0262575 (PMC8843236; doi:10.1371/journal.pone.0262575)
Supplement: S1 File — (PDF) [file pone.0262575.s001.pdf]

Citizen Science Toolkit

TABLE OF CONTENTS

1. Schedule for Citizen Science Working Group……………………………………………………2
2. Menu of Citizen Science Methods……………………………………………………………3
3. Citizen Science Activities Overview…………………………………………………………4
4. Question Bank
   4.1 Activity 1
      4.1.1 Overview……………………………………………………………………5
      4.1.2 Survey Question Bank……………………………………………………6
      4.1.3 Interview Guide……………………………………………………………10
   4.2 Activity 2
      4.2.1 Overview……………………………………………………………………14
      4.2.2 Survey Question Bank ……………………………………………………15
      4.2.3 Interview Guide……………………………………………………………16
   4.3 Activity 3
      4.3.1 Overview……………………………………………………………………19
      4.3.2 Survey Question Bank ……………………………………………………20
      4.3.3 Interview Guide……………………………………………………………21
   4.4 Activity 4
      4.4.1 Overview……………………………………………………………………24
      4.4.2 Interview Guide ……………………………………………………………25
   4.5 Activity 5
      4.5.1 Overview……………………………………………………………………28
      4.5.2 Interview Guide……………………………………………………………29

Schedule for Citizen Science Working Group

Week	Date	Duration	To Do
**1**	**Nov 1 – Nov 8**<br><br>Study kickoff	1 hour	• View orientation presentation<br>• Overview training of citizen science questions/methods<br>• Homework: Assess feasibility of selected approach
**2**	**Nov 9 – Nov 15**<br><br>Emory coach meets with each team	1 hour	• Check in regarding steps to determining feasibility<br>• Assist with data collection method
**3**	**Nov 16 – Nov 25**<br><br>Emory coach meets with each team	1 hour	• Discuss progress on data collection<br>• Trouble shoot challenges
**3-5**	**Nov 16 – Dec 4**<br><br>Data collection Period	12 hours	• Disseminate surveys<br>• Conduct interviews
**6**	**Dec 7 - 12**<br><br>Team leaders meet as a group	1.5 hours	• Turn in data, discuss additional clean up
**7-9**	**Dec 13 – Jan 3**<br><br>Hiatus [optional data collection]	Make up hours	• Can check in with coaches as needed up till Dec 20
**10**	**Jan 11-16**<br><br>Full group meeting to review findings	1.5 hours	• Shared discussion of findings with mini presentations from teams

Menu of Citizen Science Methods

Methods	Descriptions
Survey	For this activity, you would identify a group to complete an anonymous short survey. This activity could be taken on by multiple CS participants who would do fewer surveys (10 or so) within survivors (or family members) or a single CS participant could identify a large online listserv community for administering the survey.
Structured Interview	For this activity, you would identify individuals who you would interview by phone or via electronic options using a set of open-ended questions. You would be asked to keep the interviews anonymous to the researchers. Each interview would take 30-45 minutes and responses to the questions would be audio-recorded and later coded.
Personal story	For this activity, you would identify pairs of individuals (a survivor paired with a close relative) and ask them to tell their story of their relationship since the cancer diagnosis. You would be provided with probing questions to guide the story telling. The stories would be audio-recorded, later coded, and remain anonymous to the researchers.
Online panel	For this activity, you would identify 3-5 individual (all survivors or close relatives or a mix of the two) to participate in an online discussion via zoom or another electronic platform. You would pose a set of questions to the panel and the discussion would be audio-recorded and later coded. Panelists would be not be identified in any materials.
Role plays	For this activity, you would try out tools that we are developing with survivors [and relatives] and record their comments and reactions to the tool.

Citizen Science Activities Overview

Activity 1: Identify connected steps of contact that would motivate survivors to seek new information via website

We want to learn about what would motivate ovarian cancer survivors to open and read a letter they receive and visit an informational website and consider encouraging their close blood relatives to visit the website as well.

Activity 2: Obtain survivors' perspectives on contacting blood relatives

We want to learn about how survivors feel about having their close blood relatives become more aware of their risk for ovarian and other cancers, and preferred way to contact relatives.

Activity 3: Obtain relatives' perspectives on being contacted

We want to learn about how close blood relatives feel about being contacted and offered information and genetic services related to their risk for ovarian and other cancers, and how they want to be contacted.

Activity 4: Collect perspectives on information sharing within families after an ovarian cancer diagnosis

We want to learn about the barriers that survivors and families face in communicating about ovarian cancer risk so that we can help them troubleshoot these challenges. Also important to understand what has worked to help facilitate conversations within families.

Activity 5: Characterize survivors' and blood relative's information needs regarding genetic counseling and testing

In order to make the website most useful, we need to better understand what survivors and relatives would want to know about genetic counseling and testing for ovarian cancer.

Activity 1:
Identify connected steps of contact that would motivate survivors to seek new information via website

Science objective: We want to learn what would motivate ovarian cancer survivors to open and read a letter they receive and visit a website to gain new information and consider ways to reach out to their close blood relatives to encourage them to visit the website as well.

Requirements: Citizen scientists can take a quantitative or qualitative (or a combination of the two) data collection approach. The process must begin with the Georgia Cancer Registry mailing a letter to survivors of ovarian cancer. The team can also work with the coach to edit or add questions you think would be relevant.

Priority information: What steps would make it most likely for the (1) Survivor to open the letter (2) read the letter, (3) Logs on to website and (4) considers options for contacting relatives. We would also like to know about any barriers to seeking new information, under what circumstances survivors might feel coerced when approached, and any insights into whether survivors identify as a community.

Activity 1
Survey Question Bank

We will soon send recruitment letters to survivors. Below are different options we are considering to encourage survivors to contact us. For each option below, please tell us the likely you would agree to the request and how taxing it would be for you.

Please tell us how **likely** you would be to agree to each of the following activities.	Not at all likely	A little likely	Moderately likely	Very likely	Extremely likely
1. Open a letter in the mail from a state cancer registry.	1	2	3	4	5
2. Read a letter from a state cancer registry carefully.	1	2	3	4	5
3. Visit a website address provided in the letter.	1	2	3	4	5
4. Write your cell phone number on a stamped postcard and return it to us.	1	2	3	4	5
5. Return in a stamped envelope a brief survey (no more than 3 questions) about your ovarian cancer experience.	1	2	3	4	5
6. Visit a website if we offered a free promotional item such as a hat, t-shirt, scarf, face mask for doing so.	1	2	3	4	5
7. Visit a website and consider providing contact information for some or all of your close relatives who are at risk for ovarian cancer.	1	2	3	4	5
8. Visit a website to tell your personal or family cancer story.	1	2	3	4	5
9. Visit a website to get the latest information on ovarian cancer.	1	2	3	4	5
10. Visit a website to learn about ways to help your close relatives reduce their cancer risk.	1	2	3	4	5

Please tell us how **taxing or burdensome** it would be for you do the following activities.	Not at all taxing	A little taxing	Moderately taxing	Very taxing	Extremely taxing
1. Open a letter in the mail from a state cancer registry.	1	2	3	4	5
2. Read a letter from a state cancer registry carefully.	1	2	3	4	5
3. Visit a website address provided in the letter.	1	2	3	4	5
4. Write your cell phone number on a stamped postcard and return it to us.	1	2	3	4	5
5. Return in a stamped envelope a brief survey (no more than 3 questions) about your ovarian cancer experience.	1	2	3	4	5
6. Visit a website if we offered a free promotional item such as a hat, t-shirt, scarf, face mask for doing so.	1	2	3	4	5
7. Visit a website and consider providing contact information for some or all of your close relatives who are at risk for ovarian cancer.	1	2	3	4	5
8. Visit a website to tell your personal or family cancer story.	1	2	3	4	5
9. Visit a website to get the latest information on ovarian cancer	1	2	3	4	5
10. Visit a website to learn about ways to help your close relatives reduce their cancer risk.	1	2	3	4	5

We are considering different options to encourage survivors to contact us. For each of the following, please tell us how likely you would reply to an invitation if we sent you a letter that asked you:

	Not at all likely	A little likely	Moderately likely	Very likely	Extremely likely
1. Visit a URL address provided in the letter?	1	2	3	4	5
2. Write your cell phone number on a postcard and return it.	1	2	3	4	5
3. Return by mail a brief survey (no more than 3 questions) about your ovarian cancer experience?	1	2	3	4	5
4. Visit a website and consider providing contact information for some or all of your close relatives who are at risk for ovarian cancer?	1	2	3	4	5
5. Visit a website to tell your personal cancer story to be used in communications with other survivors?	1	2	3	4	5
6. Visit a website to get the latest information on ovarian cancer?	1	2	3	4	5
7. Visit a website to find out ways to help your close blood relatives reduce cancer risk?	1	2	3	4	5

How much would you trust information about ovarian cancer provided by a website hosted by the following groups?

	Not at all trusting	A little trusting	Moderately trusting	Very trusting	Extremely trusting
1. A state cancer registry	1	2	3	4	5
2. A public health research institution	1	2	3	4	5
3. A cancer research center	1	2	3	4	5
4. A community-based organization	1	2	3	4	5
5. A state public health department	1	2	3	4	5
6. Other, write in_________________	1	2	3	4	5

Activity 1
Interview Guide

GUIDELINES

- *Please be aware that you don't have to ask every question in the question bank.*
- *Please personalize the questions in the question bank and feel free to suggest questions to add that are relevant.*
- *Please use the below interview introduction script to get verbal consent from your interviewee, **before** you record the interview.*
- *The interview should stay anonymous, please use first names only during the interview.*

INTERVIEW INTRODUCTION

I am working as a "citizen scientist" on a research project at Emory's School of Public Health funded by the National Cancer Institute. I am helping researchers gain insights into the experiences of ovarian cancer survivors and their close blood relatives. The goal of the project is to encourage survivors and their close blood relatives to visit a website to learn more about their risk for ovarian and other cancers and to also consider no-cost genetic counseling service.

I'd like to ask you a few questions about what would motivate ovarian cancer survivors to open and read a letter they receive, visit an informational website, and consider encouraging their close blood relatives to visit the website as well. The interview will take about 30 minutes.

- Do you have questions about what I am asking you to help with?
- Are you willing to take part? *[if no, stop the interview]*
- May I record this interview? *[if no, stop the interview]*

INTERVIEW QUESTIONS

Please note that this guide only has the main themes to discuss with the participants and as such does not include the various prompts that may also be used (examples given for each question). Non-leading and general prompts will also be used, such as "please elaborate", "can you please tell me a little bit more about that?" and "what does that look like for you?"

1. How much have you thought about whether other [you or other] close blood relatives might also be at risk of getting cancer?

2. Think about how you identify yourself. What comes to mind first?

   [*Example: for me, I see myself as a mother, spouse, and daughter.]*

- What is it for you? Would being a survivor of ovarian cancer be on that list? Why or why not?

3. In what ways has the experience of ovarian cancer affected how you think about yourself?

4. When you are called upon to do something because you are a cancer survivor [or a relative of a cancer survivor], such as donating to or participating in something, how does that make you feel? What makes you want to help? When has an approach turned you away? Or what are some aspects of outreach that seem unfavorable to someone in your position?

5. People get mail all the time from people or organizations they don't know.
   - Can you think of a time that you opened a letter from an organization you didn't know? What was it about the letter that prompted you to open it? Would you open other letters that were like that – or is there some aspect of a letter that prompts you to open it?
   - What kind of letters do you never open? What is it about them that makes you unwilling open them or complete an enclosed request?

6. Imagine you received a letter from Georgia Cancer Registry.
   - First, is the GA Cancer Registry an organization you know? If not, would you open a letter from this organization or a cancer organization that you don't know?
   - If yes, what do you know about the GA Cancer Registry? Would you open this letter? Why or why not?
   - What key items should be on the letter to invite you to open it?

7. One thing we hope to do with this project is get people to visit a website that gives them guidance regarding how to talk about ovarian cancer to relatives who are at risk for the cancer. Would you go to such a website?
   - If yes, why?
   - If no, why not?

   [*Suggested probes depending on the answer: Do you already know about how to talk to relatives? Have you decided not to talk to relatives? If so, would anything change your mind? Does this kind of talk make you nervous or uncomfortable? If it does make you nervous, what could we put on the website to make this all easier for you?*]

8. One purpose of the webpage is to gather information. The single goal of gathering information is to know what approach you are most comfortable with when you go about contacting your relatives about their genetic or ovarian cancer risk. If you want someone with cancer expertise to contact your relatives, what kind of information would you be willing to share about yourself and family? [*Need to specify confidentiality of website and who can view it.*]

    - List type of information—e.g., names of relatives, contact information for relatives—and ask if they would be willing to share the information.

      [*Suggested probes: could follow by asking why or why not; could follow by asking what would make you willing to share the information*.]

9. We ultimately want survivors of ovarian cancer to encourage their blood-relative to visit the website. Would you ever encourage a blood-relative to visit a website that tells them about their risk for ovarian cancer?

    - If not, why would you not want your relatives to visit the website? Does it depend on the relative? What is it about the relative that makes you uncomfortable sharing the website? Does your relationship with them or their personality contribute to your potential discomfort in encouraging them?
    - What would you need to see on the website before you encouraged a blood-relative to visit the website?

10. Think back to those early days when you first found out you had ovarian cancer. Is there anything you wish you had known before learning you had ovarian cancer? Do you wish you were made aware of your risk earlier?

    - If yes, who should have done that?
    - If not, can you explain to me why not? What would have changed your mind? Unfortunately, your close blood-relatives are higher risk for ovarian cancer.
    - What do you think they should know about their risk now?
    - What advice would you give to them?

11. We are considering different options to encourage survivors to contact us.

    - What do you think might be a good way to encourage survivors to visit our website?
    - Would a letter from the Emory team be effective in encouraging them to visit our website?
    - Is there a better way? If yes, explain.
    - What about a more personalized approach? What would that look like?

Thank you very much for your time and the information you shared today.

[*End of the interview*]

After your complete the interview, please stop recording on WebEx. A copy of your interview will be automatically saved in the cloud for the study team to access.

If you have any questions, please contact Jingsong.zhao@emory.edu

Activity 2:
Obtain survivor perspectives on contacting blood relatives

Science Objective: We want to learn about how survivors feel about having their close blood relatives become more aware of their risk for ovarian and other cancers.

Requirements: Citizen scientists can take a quantitative or qualitative (or a combination of the two) data collection approach. The team can also work with the coach to edit or add questions you think would be relevant.

Priority Information needed: *We want to gain insights into how aware survivors are of their* relatives' risks for ovarian and other cancers. Additionally, how aware are they of the potential health benefits that relatives could gain from risk assessment. We want to better understand the extent to which survivors regard their relatives' contact information to be sensitive and in what contexts survivors would be willing to share contact information. We would also like to hear from survivors what might be the pros and cons of having others contact their relatives.

Activity 2:
Survey Question Bank

Below are several ways to tell contact close blood relatives about their risk for ovarian cancer. How comfortable are you with each?

	Not at all comfortable	A little comfortable	Moderately comfortable	Very comfortable	Extremely comfortable
1. Me personally contacting my relatives about their risk.	1	2	3	4	5
2. Someone with cancer expertise to contacting my relatives about their risk.	1	2	3	4	5
3. Someone who has a relative with ovarian cancer contacting my relatives about their risk.	1	2	3	4	5
4. Me personally contacting some of my relatives and someone with cancer expertise contacting other relatives.	1	2	3	4	5
5. I would not want any of my relatives contacted about their risk.	1	2	3	4	5

Activity 2:
Interview Guide

GUIDELINES

- *Please be aware that you don't have to ask every question in the question bank.*
- *Please personalize the questions in the question bank and feel free to suggest questions to add that are relevant.*
- *Please use the below interview introduction script to get verbal consent from your interviewee, **before** you record the interview.*
- *The interview should stay anonymous, please use first names only during the interview.*

INTERVIEW INTRODUCTION

I am working as a "citizen scientist" on a research project at Emory's School of Public Health funded by the National Cancer Institute. I am helping researchers gain insights into the experiences of ovarian cancer survivors and their close blood relatives. The goal of the project is to encourage survivors and their close blood relatives to visit a website to learn more about their risk for ovarian and other cancers and to also consider no-cost genetic counseling service.

I'd like to ask you a few questions about how survivors feel about having their close blood relatives become more aware of their risk for ovarian and other cancers. The interview will take about 30 minutes.

- Do you have questions about what I am asking you to help with?
- Are you willing to take part? *[if no, stop the interview]*
- May I record this interview? *[if no, stop the interview]*

INTERVIEW QUESTIONS

Please note that this guide only has the main themes to discuss with the participants and as such does not include the various prompts that may also be used (examples given for each question). Non-leading and general prompts will also be used, such as "please elaborate", "can you please tell me a little bit more about that?" and "what does that look like for you?"

1. As you think about different outreach approaches [*listed below*] for contacting relatives to give them updated information about ovarian cancer prevention options, would you be comfortable with XXXXXXX (name the approach) approach? If yes, why? If no, why not? Would you be comfortable with XXXXXXX approach? If yes, why? If no, why not?

   Outreach Approaches:

   - *Me personally contacting my relatives about their risk*
   - *Someone with cancer expertise contacting my relatives about their risk*

- *Someone who has a relative with ovarian cancer contacting my relatives about their risk*
- *Me personally contacting some of my relatives and someone with cancer expertise contacting other relatives*

2. How does it make you feel when you get letters or emails about your ovarian cancer?
    - Let's say you get an email telling you there is an informational program for cancer survivors – how would you feel? Would you be interested in going? If you would not be interested in going, why not?
    - Or let's say you get a letter from your medical team to see how you are doing and if you would call them. Would you call? How would you feel getting that letter? (e.g. upset, sad, thankful, concerned, etc.)
    - Would you have preferred to receive this request in a different way (by phone, email, etc.)? What if this letter was about an educational program and asked you to call your medical team. Would you call? Why or why not?

3. What is the last thing you remember seeing in the news or online related to ovarian cancer? Describe what you remember. What was the source of the information? Did you think the info was reliable? Why or why not?

4. What got your attention about this news? How was it different from other news you have seen about ovarian cancer? What made this news different and worth reading about? Was it the source, the way it was presented, or the information?

5. What is your most trusted source for information about ovarian cancer? Tell me about the specific source and why you trust it.

6. Are there sources of information about ovarian cancer that you don't trust? What are some examples? What about them makes you not trust them?

7. Can you describe for me the ideal way you would want to be contacted to get new information about how to prevent ovarian cancer? Use your imagination. What would be the best way possible to get you that info and who would give it to you?

    [*Probe if needed: phone call, email, letter in the mail*]

8. Do you ever think about whether other [*you or other*] close relatives might also be at risk of getting cancer? How often do you think about it (a lot, sometimes, never)?

Thank you very much for your time and the information you shared today.

[*End of the interview*]

After your complete the interview, please stop recording on WebEx. A copy of your interview will be automatically saved in the cloud for the study team to access.

If you have any questions, please contact Jingsong.zhao@emory.edu

Activity 3:
Obtain relatives' perspectives on being contacted

Science Objective: We want to learn about how close blood relatives feel about being contacted and offered information and genetic services related to their risk for ovarian and other cancers.

Requirements: Citizen scientists can take a quantitative or qualitative (or a combination of the two) data collection approach. The team can also work with the coach to edit or add questions you think would be relevant.

Priority information needed: We want to gain insights into how aware relatives are of their risks for ovarian and other cancers. Additionally, how aware are they of the potential health benefits they could gain from risk assessment. We want to better understand the extent to which relatives' feel that their contact information is sensitive and in what contexts relatives would be willing to be contacted. We would also like to hear from relatives what might be the pros and cons of different outreach strategies and willingness to visit an informational website.

Activity 3:
Survey Question Bank

How comfortable would you be with each of the following outreach approaches aimed to help you get new information about ovarian cancer prevention?

	Not at all comfortable	A little comfortable	Moderately comfortable	Very comfortable	Extremely comfortable
1. I would want my family member with ovarian cancer to contact me personally?	1	2	3	4	5
2. I would want someone with cancer expertise to contact me?	1	2	3	4	5
3. I would want someone who has a relative with ovarian cancer contact me?	1	2	3	4	5
4. I would not want to be contacted by anyone?	1	2	3	4	5

Activity 3:
Interview Guide

GUIDELINES

- *Please be aware that you don't have to ask every question in the question bank.*
- *Please personalize the questions in the question bank and feel free to suggest questions to add that are relevant.*
- *Please use the below interview introduction script to get verbal consent from your interviewee,* ***before*** *you record the interview.*
- *The interview should stay anonymous, please use first names only during the interview.*

INTERVIEW INTRODUCTION

I am working as a "citizen scientist" on a research project at Emory's School of Public Health funded by the National Cancer Institute. I am helping researchers gain insights into the experiences of ovarian cancer survivors and their close blood relatives. The goal of the project is to encourage survivors and their close blood relatives to visit a website to learn more about their risk for ovarian and other cancers and to also consider no-cost genetic counseling service.

I'd like to ask you a few questions about how close blood relatives feel about being contacted and offered information and genetic services related to their risk for ovarian and other cancers. The interview will take about 30 minutes.

- Do you have questions about what I am asking you to help with?
- Are you willing to take part? *[if no, stop the interview]*
- May I record this interview? *[if no, stop the interview]*

INTERVIEW QUESITONS

Please note that this guide only has the main themes to discuss with the participants and as such does not include the various prompts that may also be used (examples given for each question). Non-leading and general prompts will also be used, such as "please elaborate", "can you please tell me a little bit more about that?" and "what does that look like for you?"

1. As you think about different outreach approaches [*listed below*] for contacting relatives to give them updated information about ovarian cancer prevention options, would you be comfortable with XXXXXXX (name the approach) approach? If yes, why? If no, why not? Would you be comfortable with XXXXXXX approach? If yes, why? If no, why not?

   Outreach Approaches:

- *My family member with ovarian cancer contacting me personally*
- *Someone with cancer expertise contacting me*
- *Someone who has a relative with ovarian cancer contacting me*
- *Not being contacting by anyone*

2. How does it make you feel when you get letters or emails about your ovarian cancer?
   - Let’s say you get an email telling you there is an informational program for cancer survivors – how would you feel? Would you be interested in going? If you would not be interested in going, why not?
   - Or let’s say you get a letter from your medical team to see how you are doing and if you would call them. Would you call? How would you feel getting that letter? (e.g. upset, sad, thankful, concerned, etc.)
   - Would you have preferred to receive this request in a different way (by phone, email, etc.)? What if this letter was about an educational program and asked you to call your medical team. Would you call? Why or why not?

3. What is the last thing you remember seeing in the news or online related to ovarian cancer? Describe what you remember. What was the source of the information? Did you think the info was reliable? Why or why not?

4. What got your attention about this news? How was it different from other news you have seen about ovarian cancer? What made this news different and worth reading about? Was it the source, the way it was presented, or the information?

5. What is your most trusted source for information about ovarian cancer? Tell me about the specific source and why you trust it.

6. Are there sources of information about ovarian cancer that you don’t trust? What are some examples? What about them makes you not trust them?

7. Can you describe for me the ideal way you would want to be contacted to get new information about how to prevent ovarian cancer? Use your imagination. What would be the best way possible to get you that info and who would give it to you?

   [*Probe if needed: phone call, email, letter in the mail*]

8. Do you ever think about whether other [*you or other*] close relatives might also be at risk of getting cancer? How often do you think about it (a lot, sometimes, never)?

Thank you very much for your time and the information you shared today.

[*End of the interview*]

After your complete the interview, please stop recording on WebEx. A copy of your interview will be automatically saved in the cloud for the study team to access.

If you have any questions, please contact Jingsong.zhao@emory.edu

Activity 4:
Collect perspectives on information sharing within families after an ovarian cancer diagnosis

***Science Objective**:* We want to learn about the ways that survivors and families communicate about ovarian cancer risk so that we can identify and address challenges and facilitate these conversations among families.

***Requirements**:* Citizen scientists can take a qualitative data collection approach. The team can also work with the coach to edit or add questions you think would be relevant.

***Priority Information Needed**:* Specifically, we want to know how communication about ovarian cancer risk flows through the family. What are the barriers to talking about ovarian cancer and what makes helps information flow.

Activity 4: Interview Guide

GUIDELINES

- *Please be aware that you don't have to ask every question in the question bank.*
- *Please personalize the questions in the question bank and feel free to suggest questions to add that are relevant.*
- *Please use the below interview introduction script to get verbal consent from your interviewee,* ***before*** *you record the interview.*
- *The interview should stay anonymous, please use first names only during the interview.*

INTERVIEW INTRODUCTION

I am working as a "citizen scientist" on a research project at Emory's School of Public Health funded by the National Cancer Institute. I am helping researchers gain insights into the experiences of ovarian cancer survivors and their close blood relatives. The goal of the project is to encourage survivors and their close blood relatives to visit a website to learn more about their risk for ovarian and other cancers and to also consider no-cost genetic counseling service.

I'd like to ask you a few questions about the ways that survivors and families communicate about ovarian cancer risk so that we can identify and address challenges and facilitate these conversations among families. The interview will take about 30 minutes.

- Do you have questions about what I am asking you to help with?
- Are you willing to take part? *[if no, stop the interview]*
- May I record this interview? *[if no, stop the interview]*

INTERVIEW QUESTIONS

Please note that this guide only has the main themes to discuss with the participants and as such does not include the various prompts that may also be used (examples given for each question). Non-leading and general prompts will also be used, such as "please elaborate", "can you please tell me a little bit more about that?" and "what does that look like for you?"

1. I know you have been through a lot with your ovarian cancer (or with your relative's ovarian cancer), have you felt like you could talk about all that happened with other people? Or do you like to keep it to yourself? Why or why not?

2. Who have you talked the most with about your experiences? Why that person?

3. Could you tell me about a conversation you have had with a close relative about your [relative's] cancer experience? How did it go? What did you discuss? What was their reaction?
   - What was it that made it possible for you to be so open with this person? What was your conversation/communication style? [*Probe: If the participant is willing, they could be asked to do a little role play of the conversation*].

4. Have you wanted to be open with some close relative but were unable to do so? Tell me about that. What got in the way?

5. If it is okay with you, it would really help us if you could tell us your whole story. Let's start at the beginning:
   - How did you find out you [your family member] had ovarian cancer?
   - How did you get through the day or first few weeks after learning about the cancer? What coping strategies did you use?
   - Whom did you tell first about the cancer? Why did you choose to tell them?
   - What was their reaction? How did their reaction make you feel?

6. Now let's think about all the people you told. Did anyone say something to you that was especially comforting? Tell me about that.
   - How about talking to your close relatives (your siblings, your children or your parents)? How difficult was that?
   - Did you decide not to tell some relatives? Why or why not?
   - Did the topic of their risk come up in that conversation with close relatives or did it come up later during your cancer journey? If later, when? If you did talk about their potential risk, how did that go? If not, why do you think their risk didn't come up?
   - Do you think having some help from a professional would have made talking about risk easier? Why or why not?

7. Talking about risk, do you think of ovarian cancer as a shared health risk in your family? If yes, is your family doing anything about this? Could you use help? If no, describe how your family views your (or your family member's) diagnosis? Did they view it as an isolated event or some other way?

Thank you very much for your time and the information you shared today.

[*End of the interview*]

After your complete the interview, please stop recording on WebEx. A copy of your interview will be automatically saved in the cloud for the study team to access.

If you have any questions, please contact Jingsong.zhao@emory.edu

Activity 5:
Characterize survivors' and blood relative's information needs regarding genetic counseling and testing

Science Objective: Even in ideal situations where cost is not an issue, we see very low uptake of genetic counseling in the context of cancer risk. In order to make the website most useful, we need to better understand what survivors and relatives would want to know about genetic counseling and testing

Requirements: Citizen scientists can take a qualitative data collection approach. The team can also work with the coach to edit or add questions you think would be relevant.

Priority Information Needed: Specifically, we want to hear from relatives and survivors what they would see as the pros and cons of talking with a genetic counselor about their cancer risk. What do they know about genetic counseling and how does that influence their (or their relatives') interest in having genetic counseling. What information do they need to have to be informed enough to decide about genetic counseling?

Activity 5:
Interview Guide

GUIDELINES

- *Please be aware that you don't have to ask every question in the question bank.*
- *Please personalize the questions in the question bank and feel free to suggest questions to add that are relevant.*
- *Please use the below interview introduction script to get verbal consent from your interviewee, **before** you record the interview.*
- *The interview should stay anonymous, please use first names only during the interview.*

INTERVIEW INTRODUCTION

I am working as a "citizen scientist" on a research project at Emory's School of Public Health funded by the National Cancer Institute. I am helping researchers gain insights into the experiences of ovarian cancer survivors and their close blood relatives. The goal of the project is to encourage survivors and their close blood relatives to visit a website to learn more about their risk for ovarian and other cancers and to also consider no-cost genetic counseling service.

I'd like to ask you a few questions about what survivors and relatives would want to know about genetic counseling and testing. The interview will take about 30 minutes.

- Do you have questions about what I am asking you to help with?
- Are you willing to take part? *[if no, stop the interview]*
- May I record this interview? *[if no, stop the interview]*

INTERVIEW QUESTIONS

Please note that this guide only represents the main themes to be discussed with the participants and as such does not include the various prompts that may also be used (examples given for each question). Non-leading and general prompts will also be used, such as "please elaborate", "can you please tell me a little bit more about that?" and "what does that look like for you?"

1. What are your thoughts on genetic counseling? Tell me what you know about it. Have you heard of it being used to find out cancer risk? Tell me about that.

2. Have you ever had genetic counseling? If yes, how did you hear about it and what was it for? If you don't mind, please tell me how it went.

3. So let's focus on genetic counseling for ovarian cancer. Have you had genetic counseling for ovarian cancer?

   3a. FOR THOSE THAT HAVE NOT

   If you have not had genetic counseling for ovarian cancer, why not? Were you never told about it or made aware of these services?

   For those who were never told about genetic counseling for ovarian cancer. If someone suggested it now, would you meet with a genetic counselor?

   - If yes, why? What do you believe are some benefits of meeting with a genetic counselor? Also, describe for me any risks you worry about.
   - If no, what would make you reluctant to meet with the counselor? Is there anything that might make you change your mind? Describe for me what you think are the benefits, if any, of meeting with a genetic counselor? Also, describe for me any risks you worry about.

   For those who were recommended to have genetic counseling but did not

   - If you were told about it, why did you decide not to go ahead with the counseling? Can you walk me through that whole decision process that you went through?
   - Is there anything that we could do now that would change your mind? Describe for me what you think are the benefits of meeting with a genetic counselor? Also, describe for me any risks you worry about.

   3b. FOR THOSE WHO HAD GENETIC COUNSELING FOR OVARIAN CANCER

   - Why did you decide to have the counseling? Was it because of the person who recommended it? Who was it? Or did you decide to have it for other reasons? What were those reasons and why did they ultimately make you decide to have the counseling?
   - Tell me about the counseling. How did it go? What were the benefits of meeting with the counselor? If it was a negative experience, what might you have wanted to be done differently? Were there any risks you worried about?

     If yes, tell me about those risks. Based on your experience, would you recommend genetic counseling to a friend or family member? Why or why not?

4. What about Genetic Testing. Have you ever had genetic testing?

   - If yes, what was it for? How did that go for you? If it was for ovarian cancer, why did you decide to do the testing? Did a genetic counselor recommend it or did you do it for some other reason? Are you glad you did it? Do you have any regrets?
   - If no, did you think about it and then decide against it? What were the main reasons you decided against it? What would have to change for you to reconsider getting genetic testing?

5. [FOR SURVIVORS] Would you want your family members to seek genetic counseling? Why or why not?

Thank you very much for your time and the information you shared today.

[*End of the interview*]

After your complete the interview, please stop recording on WebEx. A copy of your interview will be automatically saved in the cloud for the study team to access.

If you have any questions, please contact *Jingsong.zhao@emory.edu*
